# Supplementary material for: Spatial impacts of the intercity flow of long stay international visitors based on mobile phone data in Yangtze River Delta, China
Source: PLoS One. 2023 Sep 7;18(9):e0290758. doi: 10.1371/journal.pone.0290758 (PMC10484463; doi:10.1371/journal.pone.0290758)
Supplement: Attachment — Submitted filename: response to reviewers.docx [file pone.0290758.s002.docx]

Dear Editor,

I have completed the Clinical Studies Checklist according to your requirements. It should be noted that my study did not involve any individual participants. The mobile signaling data used in this study was directly obtained from the operator of China Unicom, and the operator retains ownership of this data and implements standard regulations for its sharing and access to ensure privacy. To safeguard personal privacy, individual phone numbers were anonymized by the operator before leaving their storage facilities, and were identified with a security ID (hash code).

This has been explained in detail in the paper, and I believe that the study does not require an ethics approval application. Of course, if you think this study still requires an ethics approval, please contact me. I will try to apply and send it to you.

Therefore, in your second request, I was asked to provide additional details regarding participant consent. However, there are no actual individual participants in this study, so I find it difficult to provide information on whether the participants agree and the type of consent.

If you need any additional information related to this study, please contact me promptly. I am looking forward to your reply.

Sincerely,

MENG
